# Multi-dimensional self-esteem and substance use among Chinese adolescents

**DOI:** 10.1186/1747-597X-9-42

**Published:** 2014-10-01

**Authors:** Cynthia ST Wu, Ho Ting Wong, Carmen HM Shek, Alice Yuen Loke

**Affiliations:** School of Nursing, The Hong Kong Polytechnic University, Kowloon, China; Hong Kong Sanatorium and Hospital, Kowloon, China; GH506, School of Nursing, The Hong Kong Polytechnic University, Hung Hom, Kowloon, Hong Kong, China

**Keywords:** Alcohol, Child & adolescent health, Drugs, Smoking, Substance use, Tobacco

## Abstract

**Background:**

Substance use among adolescents has caused worldwide public health concern in recent years. Overseas studies have demonstrated an association between adolescent self-esteem and substance use, but studies within a Chinese context are limited. A study was therefore initiated to: (1) explore the 30 days prevalence of substance use (smoking, drinking, and drugs) among male and female adolescents in Hong Kong; (2) identify the significant associations between multidimensional self-esteem and gender; and (3) examine the relationship between multi-dimensional self-esteem and substance use.

**Methods:**

A self-esteem scale and the Chinese version of the global school-based student health survey were adopted. A total of 1,223 students were recruited from two mixed-gender schools and one boys’ school.

**Results:**

Among females, there was a lower 30-day prevalence of cigarette, alcohol, and drug use. They also had significantly higher peer and family self-esteem but lower sport-related self-esteem. Body image self-esteem was a predictor of alcohol use among females, while peer and school self-esteem were predictors of drug use among males.

**Conclusions:**

In summary, the findings demonstrated the influence of self-esteem to the overall well-being of adolescents. Schools could play a role in promoting physical fitness and positive relationships between adolescents and their peers, family, and schools to fulfill their physical and psychological self-esteem needs.

## Background

The primary cause of mortality and morbidity among adolescents has shifted from infectious diseases to behavioral etiologies and social interactions [1–4]. Adolescent behavior, in conjunction with psychological and social factors, often contributes to lifestyle or behavioral choices that contribute to serious health consequences [5]. Substance use is a risk behavior of adolescents that has caused worldwide public health concern in recent years, and Hong Kong is no exception to this concern [6, 7]. The trend of adolescent substance abuse is rising [8]. According to the report of the Narcotics Division in Hong Kong, the percentage breakdown of lifetime alcohol, cigarette, and drug use among secondary school students from 2004–2005 was 66.5%, 15.6%, and 3.3% respectively [6]. These respective percentages changed to 64.9%, 12.2%, and 4.3% from 2008–2009 [7]. The rate of drug use increased 1% within the last four years; while the rates of alcohol and cigarette use both decreased, the percentages remained high.

Studies suggest that peer pressure acted as a significant predictor of alcohol and cigarette use in satisfying the need for friendship [9, 10]. Poor family functionality and peer influences may push adolescents towards substance use [11]. Low self-esteem predicted substance use in adolescence [12, 13]. Adolescents with low self-esteem engaged in substance use as a way to cope with negative feelings and escape from stressors [14]. Some studies contradicted these findings and noted an insignificant association between low self-esteem and the specified risk behaviors [13, 15–17]. The inconsistencies across various studies may be due to variations in demographic groups, culture, and definitions of self-esteem. There were aspects of self-esteem that people were unwilling or unable to report [18]. The perspectives towards the concept of self-esteem and substance use might be varied in adolescents of differing cultural backgrounds and gender [19–21]. Parental support helps elevate feelings of self-worth and therefore promotes the emotional and behavioral adjustments of adolescents [22].

Wild et al. [20] also observed that the inconclusive results of the relationships between self-esteem and risk behaviors may due to the varying operational definitions of uni-dimensional self-esteem used by different researchers. Moreover, certain risk behaviors may only be related to specific types of self-esteem. In this connection, they proposed that levels of self-esteem among adolescents should be measured using a multidimensional approach with the intent to examine relationships between specific factors of self-esteem and particular risk behaviors among adolescents. Currently, the concept of multidimensional self-esteem has been widely applied on different risk behaviors such as bullying [23, 24], suicide [25], etc. [26, 27]. However, few applications of multidimensional self-esteem were related to the problem of substance use.

**Figure 1:**
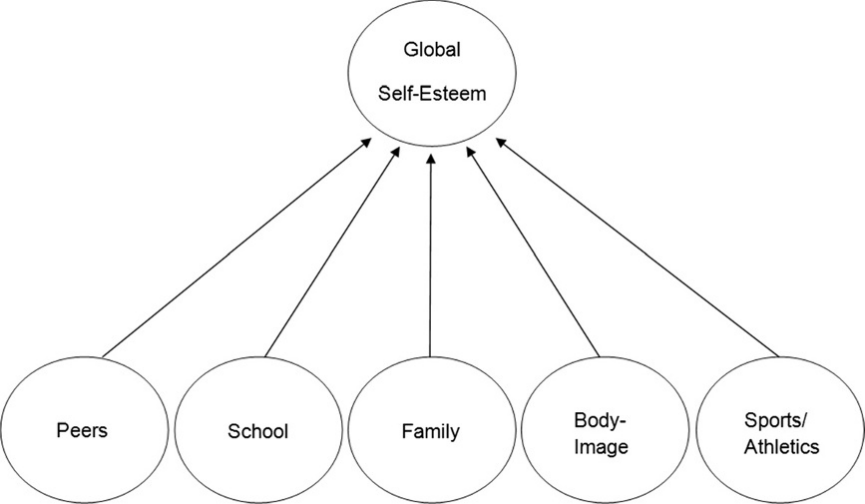
**The factor structure of multidimensional self-Esteem questionnaire.**

Among the limited studies of multidimensional self-esteem and substance use, the one conducted by Wild et al. [20] adopted the multidimensional Self-Esteem Questionnaire (SEQ) for identifying the association of different risk behaviors (including substance use). This questionnaire was first proposed by DuBois et al. [22]. It consists of 6 factors (i.e. peers, school, family, body, sports, and global self-esteem) which were grouped to be consistent with the developmental-ecological perspective. The 6 factors were structured in two layers with the factor of global self-esteem located at the higher layer and affecting the other five factors in the lower layer (Figure 1). A high R^2^ that equaled to 0.87 was obtained in their structural equation model. Wild’s Results showed that most of the self-esteem subscale scores were associated with substance use. Particularly, peer, school, and family self-esteem were related to smoking of both genders, while sports and body self-esteem were associated with male and female smoking respectively. For the case of alcohol use, peer, school, and family self-esteem were related to female alcohol use, whereas only family self-esteem was related to male alcohol use. The result of drug use was least promising, as school and body self-esteem were related to female drug use, whereas no self-esteem sub-score was associated with male drug use.

Alternatively, Barlow and Woods [28] also mentioned that the inconclusive results of the association between self-esteem and smoking could be the result of researchers using different definitions of self-esteem to identify the relationships between uni-dimensional self-esteem and smoking, but smoking may only associated with specific type of self-esteem. Hence, they investigated the relationships between multi-dimensional self-esteem and smoking among young people by using a multi-dimensional self-esteem scale with six dimensions (i.e. scholastic competence, social acceptance, athletic competence, physical appearance, behavioral conduct, and global self-worth). Their results showed that smoking was generally associated with low self-esteem for children 10–11 years old. Particularly, scholastic competence, physical appearance and behavioral conduct were significantly lower among 10-year-old children who tried smoking, while the factor of scholastic competence was no longer significant for 11-year-old children who tried smoking.

Although multidimensional self-esteem and substance use varies depending on the culture, there are very few studies to support this supposition. Therefore, this study was performed to examine differences between self-esteem factors and substance use among Chinese adolescents.

The aims of this study were: (1) to explore the 30 day prevalence of substance use (smoking, drinking, and drug use) among male and female adolescents in Hong Kong; (2) to identify the significant associations between multidimensional self-esteem and gender; and (3) to examine the relationship between multi-dimensional self-esteem and substance use.

Instead of merely confirming low self-esteem is related to substance use, clarifying the relationships between specific self-esteem types and substance use among different groups of people is important. Findings from this study (and these types of studies) can help health educators craft education programs for specific audiences. DuBois’s multi-dimensional SEQ’s self-esteem domains are related to different aspects of human life. Delivering education programs that focus on irrelevant aspects will simply waste resources.

## Methods

### Participants

Three secondary schools accepted the research invitation. The school principals at these schools were contacted and permission was obtained. Negotiations with the school principals and class teachers were conducted to select the dates to distribute the questionnaires to students in classes (to avoid conflicts with scheduled examinations). An information sheet written in simple language that provided a brief explanation about the aims of the study and a consent form were both sent to all students and their parents prior to the survey. Ethics approval for this study was granted by the Human Subjects Ethics Committee Board of the research institution.

### Instruments

The measures consist of a self-developed questionnaire for basic demographic information and two validated tools—the self-esteem scale and substance use measure—to solicit information on adolescent multidimensional self-esteem and substance use respectively.

#### Self-esteem

The multidimensional self-esteem questionnaire (SEQ) developed by DuBois et al. was adopted [22] for this study. The SEQ is divided into six subscales. One subscale, global self-worth, directly assesses overall feelings of self-worth and consists of 8 items, such as “I am happy with the way I can do most things.” The other five subscales are social competence, academic competence, family bonding and recognition, body image, and athletic competence.

The social competence subscale consisted of 8 items like “I am as good as I want to be at making new friends.” The academic competence contained 7 items; for example one of the items is, “I am as good a student as I would like to be.” Family bonding and recognition include 8 items such as “I am happy about how much my family likes me.” Body image is related to perception about physical appearance and consisted of 4 items such as “I am happy with the way I look.” Athletic competence includes 6 items like “I wish I was better at sports/physical activities.”

A total of 42 items rated on a 4-point scale ranging from strongly disagree to strongly agree (scores 1 to 4) were used. In order to guard against possible response bias, ten items were negatively worded and reversely scored, so that the higher the score, the higher the self-esteem that an adolescent had [22]. In this study, one of the items “I get too many bad grades on my report cards” was discarded since the concept of “report cards” may not be suitable for use in Hong Kong. Hence, 41 items remained [22].

#### Substance use

The global school-based student health survey (GSHS) developed by the World Health Organization was adopted for this study to assess student substance use behaviors [29]. It is a self-administered, school-based survey used primarily with young people. The Chinese version of the GSHS questionnaire has been used in various studies and found to be reliable and valid for young people 11 and older [30–32]. It has a Kappa reliability test range of 61% to 100% [30]. In this study, a student was considered to have used a substance (i.e., cigarettes, alcohol, or drugs) if he or she had used it within the 30 days prior to completing the survey.

Permission was obtained from the author of the SEQ for the research team to translate the scale into Chinese. The Chinese version was then translated back into English by a bilingual language expert who had neither seen nor was familiar with the English original. This English back-translation was compared to the English original by two health educators and a nursing scholar with expert knowledge in educational language and public health language respectively. Face validity of the questionnaire contents were then reviewed by two nursing research experts. Minor discrepancies were discussed and resolved, and appropriate amendments were made.

The final questionnaire consisting of the Chinese version of the SEQ and GSHS was tested among 10 secondary school students over a 2-week interval to check that it was intelligible and acceptable. These students were not part of the main study sample. The coefficient alphas for each subscale ranged from 0.81 to 0.91; the test-retest correlation of the self-report questionnaire over a 2-week interval was 0.89.

### Data analysis

Descriptive statistics were used for analyzing the demographic background of the subjects and the 30-day prevalence of substance use. ANCOVA with the factor of year of study and religion being controlled was used to examine the gender differences on the self-esteem subscales. Moreover, the difference between specific types of substance users and non-users were also compared with special attention on the interaction effect of gender. Subgroup analysis by gender was done when the p-value of the interaction effect was smaller than 0.1. Finally, logistic regression was used with a forward (Wald) variable selection method to determine the predictors of substance use by gender. Bivariate analysis (threshold value 0.1) was used for deciding which of the variables in Table 1 would be considered in the logistic regression model. All data analysis was completed using SPSS 18, and the statistical significance was set at 0.05.

**Table 1:** **Demographics of secondary school students**

Demographics		Male		Female		Total	
		n	(%)	n	(%)	N	(%)
Age	11-14	518	(57.9)	207	(62.9)	725	(59.3)
	15-16	354	(39.6)	115	(35.0)	469	(38.3)
	>16	22	(2.5)	7	(2.1)	29	(2.4)
Year of study^#^	Form 1	273	(30.5)	127	(38.6)	400	(32.7)
	Form 2	322	(36.0)	91	(27.7)	413	(33.8)
	Form 3	299	(33.4)	111	(33.7)	410	(33.5)
Religion	Yes	224	(27.4)	122	(37.1)	366	(30.0)
	No	648	(72.6)	207	(62.9)	855	(70.0)
Total		894	(100)	329	(100)	1223	(100)

^#^Form 1 to 3 is equivalent to year 7 to 9 in US education system.

## Results

The demographic characteristics and substance use of the secondary school students are summarized in Table 1. A total of 1,223 students were recruited from two mixed-gender schools and one boys’ school. The majority of students (59.3%) were 11 to 14 years old, and about one-quarter (26.9%) of the subjects were female. The students were distributed evenly in Form 1 to Form 3 with around one-third in each Form (Form 1 to 3 is equivalent to year 7 to 9 in US education system). The majority had no religious affiliation (70%).

Among the three types of substances, drug use was the least frequently reported. The overall 30-day prevalence of drug use among adolescents was 1.3%, while the figures for males and females were 1.6% and 0.6% respectively. Alcohol use was the most common substance (21.0%), with 22.8% and 16.1% for males and females respectively. An overall 6.9% of adolescents were smokers, with 7.2% and 6.1% of males and females, respectively, reported that they had smoked.

Most students with substance use reported they were 12 to 13 years old when they first used cigarettes (39.3%) or alcohol (26.2%). In contrast, most (40%) first experimented with drugs when they were younger than 8 years old. During the 30 days prior to taking the survey, the majority of students had used cigarettes (34.5%) or alcohol (49.6%) on 1 or 2 days, whereas the majority of drug use was 3 to 5 days (43.8%) followed by 1 or 2 days (37.5%) Table 2.

**Table 2:** **30-day prevalence of substance use**

			Cigarette use		Alcohol use		Drug use	
			n	(%)	n	(%)	n	(%)
Substance use 30-day prevalence by gender		Male	64	(7.2)^#^	204	(22.8)^#^	14	(1.6)^#^
		Female	20	(6.1)^#^	53	(16.1)^#^	2	(0.6)^#^
		Total	84	(6.9)^#^	257	(21.0)^#^	16	(1.3)^#^
GSHS Questions	Age at first substance use^†^	≦7	9	(10.7)	44	(17.2)	6	(40.0)
		8-9	11	(13.1)	42	(16.4)	4	(26.7)
		10-11	10	(11.9)	54	(21.1)	0	(0.0)
		12-13	33	(39.3)	67	(26.2)	2	(13.3)
		14-15	14	(16.7)	38	(14.8)	1	(6.7)
		≧16	7	(8.3)	11	(4.3)	2	(13.3)
		Total	84	(100)	256	(100)	15	(100)
	Substance use during last 30 days	1-2 days	29	(34.5)	127	(49.4)	6	(37.5)
		3-5 days	9	(10.7)	45	(17.5)	7	(43.8)
		6-9 days	5	(6.0)	18	(7.0)	1	(6.3)
		10-19 days	14	(16.7)	17	(6.6)	0	(0.0)
		20-29 days	8	(9.5)	13	(5.1)	0	(0.0)
		30 days	19	(22.6)	37	(14.4)	2	(12.5)
		Total	84	(100)	257	(100)	16	(100)

^#^The percentages are the 30 days prevalence.

^†^Only students with substance use were included.

### Self-esteem and gender

Table 3 shows the mean score of self-esteem factors by gender. The female students had significantly higher mean scores for both peer (male: 22.45, SD = 3.96 vs female: 24.21, SD = 2.95; *p* < 0.01) and family factors (male: 22.45, SD = 3.57 vs female: 23.33, SD = 3.35; *p* < 0.001) than males. In contrast, a significantly lower score was observed on the factor of sports (male: 17.56, SD = 3.40 vs female: 17.19, SD = 3.07; *p* < 0.05). No other significant difference between genders was observed on other self-esteem subscales.

**Table 3:** **Self-esteem subscale by gender**

Self-esteem subscale	Male (n = 894)		Female (n = 329)		Total (N = 1223)		p-value
	Mean	(SD)	Mean	(SD)	Mean (SD)		
Peers	22.45	(3.96)	24.21	(2.95)	23.66	(3.73)	0.003**
School	18.52	(3.61)	18.20	(3.16)	18.44	(3.50)	0.114
Family	22.45	(3.57)	23.33	(3.35)	22.68	(3.53)	0.001***
Body image	10.84	(2.04)	10.64	(1.78)	10.79	(1.98)	0.080
Sports	17.56	(3.40)	17.19	(3.07)	17.46	(3.32)	0.028*
Global	22.47	(3.17)	22.53	(2.67)	22.49	(3.05)	0.947

Statistical test: ANCOVA; Controlled variables: education, and religion; df: Intercept = 1, Religion = 1, Education = 1, Gender = 1, Error = 1217; Total = 1221; *p <0.05; **p < 0.01; ***p < 0.001.

### Self-esteem and substance use

Table 4 shows the mean difference in self-esteem levels among substance users and non-users, stratified by gender and type of substance. The female non-alcohol users had a significantly higher level of school (user: 17.40, SD = 3.05 vs non-user: 18.36, SD = 3.17; *p* < 0.05) and body image self-esteem (user: 10.11, SD = 1.76 vs non-user: 10.74, SD = 1.77; *p* < 0.05) than the alcohol users. Similarly, the male non-drug users had higher peer self-esteem than the drug users (user: 20.86, SD = 6.09 vs non-user: 23.49, SD = 3.91; *p* < 0.05).

**Table 4:** **Comparison between substance users and non-users on self-esteem subscales**

SE subscale	Gender	Cigarette user		Non-cigarette user		p-value
		Mean	(SD)	Mean	(SD)	
**Peers**	**Male**	23.08	4.18	23.48	3.95	0.566
	**Female**	24.00	2.85	24.23	2.96	
**School**	**Male**	18.52	3.85	18.52	3.59	0.168
	**Female**	17.00	3.49	18.28	3.13	
**Family**	**Male**	22.22	4.10	22.46	3.53	0.886
	**Female**	23.45	3.53	23.32	3.34	
**Body image**	**Male**	10.98	2.07	10.83	2.04	0.752
	**Female**	10.35	1.95	10.66	1.77	
**Sports**	**Male**	17.77	3.39	17.55	3.40	0.740
	**Female**	16.65	3.01	17.22	3.07	
**Global**	**Male**	22.34	3.05	22.48	3.18	0.884
	**Female**	22.75	2.17	22.51	2.71	
**SE subscale**	**Gender**	**Alcohol user**		**Non-alcohol user**		**p-value**
		**Mean**	**(SD)**	**Mean**	**(SD)**	
**Peers**	**Male**	23.57	4.04	23.42	3.94	0.833
	**Female**	23.89	2.41	24.28	3.04	
**School**	**Male**	18.73	3.70	18.46	3.59	0.356
	**Female**	17.40	3.05	18.36	3.17	0.042*
**Family**	**Male**	22.38	3.56	22.47	3.58	0.610
	**Female**	23.21	3.34	23.36	3.35	
**Body image**	**Male**	10.92	1.97	10.82	2.06	0.496
	**Female**	10.11	1.76	10.74	1.77	0.022*
**Sports**	**Male**	17.87	3.27	17.47	3.43	0.900
	**Female**	16.74	2.65	17.27	3.14	
**Global**	**Male**	22.61	3.18	22.43	3.17	0.671
	**Female**	22.21	2.32	22.59	2.74	
**SE subscale**	**Gender**	**Drug user**		**Non-drug user**		**p-value**
		**Mean**	**(SD)**	**Mean**	**(SD)**	
**Peers**	**Male**	20.86	6.09	23.49	3.91	0.024*
	**Female**	26.50	0.71	24.20	2.95	0.195
**School**	**Male**	18.79	5.25	18.52	3.58	0.942
	**Female**	18.00	7.07	18.20	3.15	
**Family**	**Male**	22.50	4.60	22.45	3.56	0.804
	**Female**	22.50	4.95	23.34	3.35	
**Body image**	**Male**	10.50	3.39	10.85	2.02	0.522
	**Female**	10.00	5.66	10.65	1.75	
**Sports**	**Male**	16.79	4.26	17.58	3.38	0.704
	**Female**	18.50	3.54	17.18	3.07	
**Global**	**Male**	22.07	4.92	22.48	3.14	0.906
	**Female**	22.50	4.95	22.53	2.67	

Statistical test: ANCOVA; Controlled variables: gender, education, and religion; df: Intercept = 1, Religion = 1, Education = 1, Gender = 1, Substance use = 1, Substance use x Gender = 1, Error = 1215; Total = 1221; *p <0.05.

### Predictors of substance use intention

Table 5 shows results of the logistic regression for finding the predictors of substance use intention by gender and type of substance. Results show that only male drug users and female alcohol users were associated with the self-esteem subscales but no other groups. Male drug users were associated with the peer and school self-esteem subscales, with odd ratios equal to 0.78 (95% CI: 0.67–0.90; *p* < 0.01) and 1.24 (95% CI: 1.03–1.50; *p* < 0.05) respectively. The female alcohol users were associated with the body image self-esteem subscale (OR: 0.82; 95% CI: 0.69–0.98; *p* < 0.05) after controlling the effects of education level (i.e. Form) and religion.

**Table 5:** **Predictors of substance use** ^**†**^

Dependent variable	Predictors	Odds-ratio	95% CI	p-value	VIF	df
Male drug users^§^ (N = 894)	Peers	0.78	(0.67, 0.90)	0.001**	1.56	1
	School	1.24	(1.03, 1.50)	0.023*	1.56	1
Female alcohol users (N = 329)	Form	1.49	(1.04, 2.13)	0.029*	1.01	1
	Religion^#^	1.86	(1.02, 3.41)	0.044*	1.00	1
	Body	0.82	(0.69, 0.98)	0.029*	1.01	1

Statistical test: Logistic regression; ^#^Religion: Yes = 1, No = 0; ^†^Insignificant regression models were omitted, and the sensitivity and specificity analysis is not provided due to sample size limitation; ^§^The factor of age was considered but finally removed by the variable selection method from the regression model. *p <0.05; **p < 0.01.

## Discussion

Males were more likely than females to have used substances, including cigarettes, alcohol, and drugs. Self-esteem factors had a role in substance use, but the inconsistent results reported in previous studies suggested that there were missing variables in mediating the relationship between self-esteem and substance use [19, 33]. In addition, little research has analyzed the gender effect on the relationship between self-esteem and substance use. The results of our study suggest that substance use was more strongly related to certain domains of self-esteem in a specific gender.

Females and males had close mean scores within each of the self-esteem subscales. However, self-esteem scores of peers and family were significantly higher for females than males. This may indicate that female adolescents associated self-worth more with perceptions of peers and family than males.

The lower school self-esteem mean score was associated with alcohol use in females. As suggested by Wild et al. [20], females might have more worries than males about their academic achievement, such as class placement, homework, and test grades, and were more prone to substance use due to this burden. The findings of this study were consistent with the studies conducted by Kawabata et al. and Wild et al. [9, 20]. The results show that low school self-esteem and substance use among adolescents were strongly correlated. However, it contrasted with findings that school self-esteem was unrelated to substance use behavior among adolescents [34]. These inconsistencies might be attributable to cultural variations in the perception of academic achievement. In Chinese culture, academic achievement is often an indicator of value development among adolescents [35]. This might have a particular influence on the school self-esteem of adolescents.

Low body image self-esteem was significantly associated with female alcohol users, but such significance did not exist within the male group. Commercials and advertisements in Hong Kong frequently emphasize thinness as a quality of attractiveness, resulting in social approval and success. Peer influence of body image may lead to substance use in female adolescents [36, 37]. Those female adolescents who felt dissatisfied with their appearance react more negatively towards body fat and weight gain than male adolescents do [38]. Body dissatisfaction placed female adolescents at risk for substance use as a method of weight management control [13, 39]. Health care interventions were indicated for those adolescents who used substances as a method of improving their body image.

With regard to drug use, the male non-drug user group showed significantly higher peer self-esteem when compared to the male drug-user group. As we expect that the majority of the peer group was non-drug users, students having higher peer self-esteem means having a better relationship with the majority of non-drug users group, which resulted in the positive effect from them.

According to the results of logistic regression analysis, lower peer and higher school self-esteem will increase the likelihood of drug use among male students. The result of peer self-esteem was expected, as discussed in the previous paragraph. However, the result of higher school self-esteem as a predictor of drug use may contradict what was found about alcohol use among female students. The inconsistent result may be due to the rarity of drug use, which significantly lowers the number of male students in this study who use drugs. Nevertheless, the results also suggest that in addition to traditional health education programs aimed at increasing student knowledge about the negative effects of drug use and how to reject the temptation, teamwork and cooperative exercises could be introduced into the programs to improve friendships. Moreover, the programs themselves could also be incorporated into curriculum so that efforts to increase knowledge about the hazards of drug use can be linked with student academic assessment results. In this way, drug use education programs will not contradict students’ academic performance.

Alternatively, the results of the logistic regression also showed that body image self-esteem is a predictor of alcohol use among females. There was a greater likelihood of alcohol use among female students with lower body image self-esteem. The results could be because alcohol is a risk factor that contributes to overweight and obesity in adolescents [40]. Hence, such knowledge could be included in health education programs if the main audiences are female students.

Overall, the significant predictors found in the logistic regression analysis were considered as having effect sizes which are large enough. The odds-ratios in Table 5 were classified as having medium size except for the self-esteem subscales predictors (i.e. peers, school, and body self-esteem) [41]. As the classification is actually for binary predictors, the small effect sizes obtained for self-esteem subscales are totally acceptable. It is because the variation of the self-esteem subscales predictors’ value is much larger than the binary predictors which only have two possible values.

### Limitations

The authors acknowledge the sampling limitation of this study. First, there was potential for response bias with the use of a self-report questionnaire, particularly when asking subjective questions about health status [42]. The self-reported data may affect the objectivity of the findings. Second, sampling bias due to the use of convenience sampling may exist. The sample might not represent the entire population due to the selection of the sample schools was affected by accessibility during the research period. The participants were recruited from two mixed-gender secondary schools and one boys’ secondary school; this caused uneven distribution of the participants’ gender. The covariate omitted in this research (e.g. students’ academic results, parents’ education level, and family income) may also affect the results in this study. Finally, although the sample size (N = 1,223) of this research is large enough for prevalence estimation [43], it may not be large enough for the logistic regression analysis [44]. Some researchers suggested that 10 cases are required for each predictor with a minimum total sample size of 100 [44]. Hence, the sample size may not be large enough for the regression analysis of group of drug use which only has a few cases. As the consequence of insufficiently large sample size is the decrease of the power of the regression model, the significant independent variables found in this study were still valid. In the future, a larger study (more than 10,000) could be conducted based on knowledge gained from this study.

## Conclusions

Most previous studies on adolescent self-esteem adopted the uni-dimensional measure of global self-esteem. This study aimed to investigate from the perspective of multidimensional measurement and provide a broader perspective in assessing adolescent behavior regarding substance use. Specific self-esteem factors can be used to guide a more structured and focused health program for adolescent development. Intrinsic student motivation regarding academic performance and achievement in school activities could be reinforced by introducing self-initiated, after-school activities. Schools could also play the role of promoting physical fitness and positive relationships between adolescents and their peers, family, and schools, in order to fulfill their self-esteem needs, both physical and psychological. Actually, the school was suggested as a viable setting for conducting brief interventions for drug abusing teenagers [45].

In summary, findings from the current study demonstrate the importance of self-esteem for the overall well-being of adolescents. More specifically, peer, school, and body image self-esteem factor scores were lower in certain substance user groups. The adoption of a multidimensional self-esteem framework could provide a more sophisticated measure of self-esteem, leading to more extensive interpretations when compared with a one-dimensional view. Future studies in this area may consider the use of the multidimensional self-esteem model in a more extensive population. Finally, this study also provides insight about enhancing adolescent self-development in the context of peers, family, school, and global settings.
